# Integrating Cardiac Biomarkers and Electrocardiogram in Pulmonary Embolism Prognosis

**DOI:** 10.7759/cureus.53505

**Published:** 2024-02-03

**Authors:** Pawel Borkowski, Nikita Singh, Natalia Borkowska, Shaunak Mangeshkar, Natalia Nazarenko

**Affiliations:** 1 Internal Medicine, Albert Einstein College of Medicine, Jacobi Medical Center, New York, USA; 2 Pediatrics, Samodzielny Publiczny Zakład Opieki Zdrowotnej (SPZOZ), Krotoszyn, POL

**Keywords:** risk assessment, troponin, b-type natriuretic peptide, biomarker, pulmonary embolism

## Abstract

Pulmonary embolism (PE) represents a significant clinical challenge that substantially impacts healthcare systems. This case report focuses on the nuances of risk stratification in PE, highlighted through the presentation of a 64-year-old female patient. The uniqueness of this case lies in the patient’s atypical presentation, where decreased exercise tolerance was the sole symptom leading to the diagnosis of PE. The patient was found to have new-onset atrial fibrillation, elevated levels of N-terminal pro-brain natriuretic peptide (NT-proBNP), and signs of right ventricular strain on imaging. This scenario underscores the necessity for a comprehensive assessment in PE cases, particularly when classic symptoms (e.g., tachycardia, shortness of breath, chest pain) are absent. We explore the incidence of PE in patients diagnosed with deep vein thrombosis, examining the critical role of cardiac biomarkers, including B-type natriuretic peptide, NT-proBNP, and troponins, in prognostication and their potential use in risk assessment tools for PE patients. Additionally, the significance of electrocardiogram evaluation in these patients and its role in risk stratification is thoroughly assessed.

## Introduction

Venous thromboembolism (VTE), which includes deep vein thrombosis (DVT) and pulmonary embolism (PE), has a yearly occurrence rate of approximately 1 per 1,000 individuals [1]. A significant proportion of DVT patients also have PE, and vice versa, although the exact prevalence varies widely across studies. It is estimated that 33-73% of patients with DVT also have a concurrent PE [2-4]. It is estimated that 51.2-97% of patients with PE also have a concurrent DVT [5-7]. This variability is attributed to differences in study populations and methodologies, e.g., the inclusion or exclusion of certain types of DVT.

While the D-dimer assay is conventionally utilized to exclude PE in patients with a low clinical probability, this study focuses on biomarkers used in risk stratification rather than diagnosis. Specifically, B-type natriuretic peptide (BNP), N-terminal pro-brain natriuretic peptide (NT-proBNP), and the troponin complex are highlighted. These biomarkers, released into the circulation in response to myocardial stress and injury, are relevant in PE [8,9]. The synthesis of research involving over 5,000 patients has established the essential role of BNP, NT-proBNP, and troponin in risk stratification for PE. These biomarkers correlate with increased short-term mortality, serious adverse events, and right ventricular dysfunction (RVD) in acute PE cases. This evidence positions these biomarkers as essential tools in risk stratification and predicting clinical outcomes in PE patients.

Furthermore, the authors explore the intricate relationship between PE and cardiac arrhythmias, specifically emphasizing atrial fibrillation (AF). The complex interplay between PE and arrhythmias, particularly AF, is critical, as AF has been linked to elevated mortality rates and adverse outcomes in patients with PE. Despite its limited role in diagnosing PE due to non-specific changes, an electrocardiogram (EKG) is crucial for risk stratification.

## Case presentation

A 64-year-old female with a history of hypertension presented to the emergency department (ED) for chronic right knee pain following trauma to the area several months ago. She took ramipril and nebivolol for her hypertension. Her vital signs were a temperature of 36.6°C, blood pressure of 131/83 mmHg, pulse rate of 67 beats/minute, and respiratory rate of 18 breaths/minute. Pulse oximetry showed a blood oxygen saturation of 98%. Physical examination revealed irregular heart rhythm, clear lung sounds, soft abdomen, crepitation and tenderness in the right knee, and tenderness in the left posterior calf with a positive Homan’s sign. A complete blood count, basic metabolic panel, and inflammatory markers were normal. An X-ray of the right knee revealed no acute fractures or dislocations. Upon further questioning, the patient reported a recent long flight, leading to ordering a venous duplex ultrasound of the lower extremities, which diagnosed left-sided DVT. She was administered a therapeutic dose of enoxaparin and admitted to the internal medicine department for further management.

**Figure 1:**
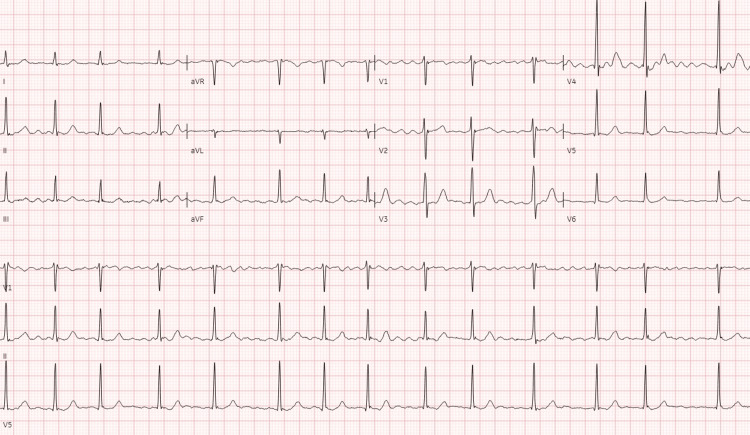
Electrocardiogram showing atrial fibrillation, heart rate of 82 beats/minute, normal axis, and incomplete right bundle branch block.

**Figure 2:**
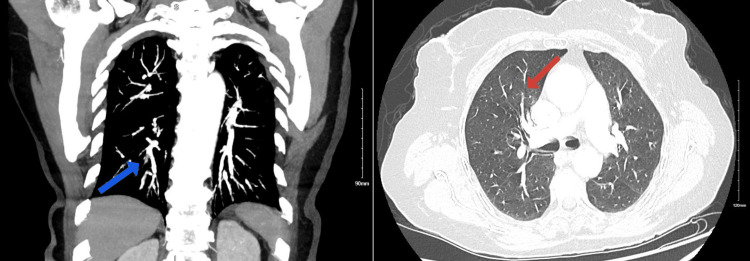
CT pulmonary angiogram showing filling defects in the subsegmental branch of the right lower lobe (blue arrow) and subsegmental branch of the right upper lobe (red arrow).

**Figure 3:**
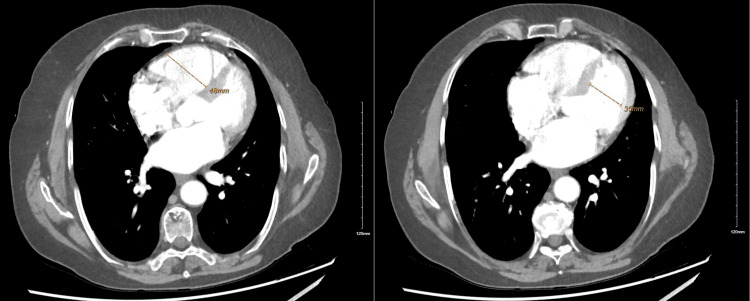
CT pulmonary angiogram. The right ventricle (RV) maximum diameter is 48 mm. The left ventricle (LV) maximum diameter is 35 mm. The ratio of RV to LV is 1.4.

The patient, after admission, reported decreased exercise tolerance, experiencing dyspnea even with minimal exertion. She had no significant cardiac history. Laboratory tests showed normal troponin I and thyroid-stimulating hormone levels but elevated NT-proBNP at 2,361 pg/mL. EKG revealed new-onset AF and incomplete right bundle branch block with a heart rate of 82 beats/minute (Figure 1). Chest X-ray was normal, and she remained hemodynamically stable. Given her symptoms, AF, and elevated NT-proBNP, a CT pulmonary angiogram was conducted, revealing acute PE in subsegmental branches of the left lower and left upper lobes with signs of right ventricle (RV) strain (right ventricle-to-left ventricle diameter ratio of 1.4) (Figures 2, 3). The patient was started on a heparin drip. Echocardiography showed an ejection fraction of 69%, no RV strain, normal right atrium size, and no tricuspid regurgitation. Cardiology service recommended against invasive procedures, transitioning her to apixaban and discharging her with instructions for close follow-ups.

## Discussion

VTE stratification requires evaluating cardiac biomarkers, namely, BNP, NT-proBNP, and the troponin complex. BNP and NT-proBNP are produced and secreted by the ventricular myocardium into the bloodstream in response to myocardial wall stress as a pro-hormone, which is then cleaved into a biologically active BNP and a biologically inactive NT-proBNP [10,11]. BNP has a shorter half-life (20 minutes) than NT-proBNP (120 minutes), resulting in NT-proBNP serum values being roughly six times higher despite both being released in equimolar quantities. The troponin complex, comprising troponin C (cTnC), troponin I (cTnI), and troponin T (cTnT), is integral for muscle contraction, with its subtypes, cTnI and cTnT, serving as markers for cardiac myocyte injury [12]. PE obstructs the pulmonary artery, leading to increased pulmonary vascular resistance and subsequent myocardial shear stress on the right ventricle, potentially resulting in acute right ventricular dilatation and myocardial ischemia [8,9]. This scenario elevates serum levels of BNP/NT‐proBNP and troponin, respectively. The aggregated data from various studies presents a clear consensus on the utility of BNP/NT-proBNP and troponin levels as reliable tools for prognostication, risk stratification, and predicting clinical outcomes in patients with PE.

In a comprehensive meta-analysis by Coutance et al., involving 12 studies and 868 patients, elevated levels of BNP and NT-proBNP were associated with increased short-term (in-hospital or up to 40-day) all-cause mortality (odds ratio (OR) = 6.57, 95% confidence interval (CI) 3.11-13.91) and serious adverse events such as shock, resuscitation, mechanical ventilation, and thrombolysis (OR = 7.47, 95% CI = 4.2-13.15) [13]. Similarly, a meta-analysis by Klok et al., encompassing 13 studies with 1,132 patients, demonstrated a heightened 30-day mortality risk (OR = 7.6, 95% CI = 3.4-17) and complicated in-hospital course (OR = 6.8; 95% CI = 4.4-10) in patients with elevated BNP or NT-proBNP levels [14]. However, both studies used diverse assays and retrospectively established cut-off points, making it challenging to determine an optimal cut-off for BNP/NT-proBNP tests due to a lack of data for receiver operating characteristic curve analysis. Limited research was conducted to compare BNP with NT-proBNP in predicting adverse clinical outcomes. A systematic review by Cavallazzi et al., involving 800 patients, evaluated both biomarkers in the context of acute PE [10]. It was found that both BNP and NT-proBNP correlate with the diagnosis of RVD and significantly predict all-cause in-hospital or short-term mortality in PE patients. The aggregated diagnostic OR for RVD detection was 39.45 (95% CI = 15.54-100.12) for BNP and 24.73 (95% CI = 2.02-302.37) for NT-proBNP. The combined OR for predicting mortality was 6 (95% CI = 1.31-27.43) for BNP and 16.12 (95% CI = 3.1-83.68) for NT-proBNP. This study also established clinical cut-off points of 100 pg/mL for BNP and 600 ng/L for NT-proBNP, offering valuable benchmarks for clinical application. Additionally, a study by Lankeit et al. involving 688 patients determined a similar NT-proBNP cut-off value of 600 pg/mL, demonstrating optimal prognostic efficiency with 86% sensitivity and 50% specificity [15]. In a substantial meta-analysis involving 1,985 patients, Becattini et al. found a strong association between increased troponin concentrations and heightened risk of short-term mortality (OR = 5.24, 95% CI = 3.28-8.38) and adverse clinical events, including shock, resuscitation, mechanical ventilation, and thrombolysis (OR = 7.03, 95% CI = 2.42-20.43) [16]. Additionally, the data supports the role of troponin in augmenting other risk stratification tools, such as the simplified Pulmonary Embolism Severity Index (sPESI). For example, Lankeit et al., using a high-sensitivity troponin T (hsTnT) cut-off value of 14 pg/mL, identified its association with early death or complications in PE patients (OR = 4.97, 95% CI = 1.71-14.43) [17]. When integrated with sPESI, the study revealed that patients with a sPESI of 0 and baseline hsTnT levels below 14 pg/mL exhibited a significant 42% reduction in mortality risk (hazard ratio = 0.58, CI = 0.01-0.42). In summary, cardiac biomarkers offer high negative predictive value that facilitates identifying patients likely to have a stable hospital course. Consequently, patients with PE exhibiting elevated cardiac biomarkers should be subjected to further echocardiographic evaluation to categorize them into low-risk or high-risk groups, thereby informing the choice between non-invasive and invasive management strategies [18]. Additionally, emerging evidence points to cardiac biomarkers playing a key role in enhancing PE risk stratification tools in the future.

PE and arrhythmias exhibit a complex, bidirectional relationship, particularly AF and atrial flutter (AFL). PE can induce arrhythmias through right-sided pressure overload or inflammatory cytokine effects, while AF/AFL may promote clot formation in the right atrium, elevating PE risk [19]. EKG manifestations in PE patients vary from normal EKGs to diverse abnormalities, including rhythm and conduction disturbances, QRS complex axis orientation changes, and alterations in the P wave, QRS complex, and ST-segment/T-wave shapes. Common EKG findings in PE include sinus tachycardia, sinus bradycardia, AF, AFL, the S1Q3T3 (S waves in lead I, Q waves in lead III, T-wave inversion in lead III) pattern, P pulmonale, right axis deviation, complete or incomplete right bundle branch block, right ventricular strain pattern, atrioventricular conduction abnormalities, ST-segment depression, and T-wave inversion [20]. This review delves into the prognostic significance of AF in PE, noting that AF may occur during the acute or recovery phases of PE [19]. A substantial study by Bikdeli et al., involving 16,497 patients, found that individuals with PE and preexisting AF had a higher risk of all-cause mortality (OR = 1.91, 95% CI = 1.57-2.32). Additionally, patients with PE and new-onset AF faced an even higher likelihood of 90-day all-cause mortality (OR = 2.28, 95% CI = 1.75-2.97) [21]. This underscores the profound impact of AF in PE prognosis. In a comprehensive retrospective study by Ng et al., which included 1,142 patients with a mean follow-up of 5.0 ± 3.7 years, the mortality rates (total mortality was 42% (n = 478)) were significantly different across three groups: those without AF, those with preexisting AF, and those who developed AF post-PE, with rates of 35% (n = 283), 59% (n = 119), and 60% (n = 76), respectively [22]. The findings highlight AF as a significant prognostic tool in patients with PE, particularly in the recovery phase. Early detection and management of AF can significantly improve clinical outcomes, stressing the need for AF screening in PE management. Additionally, EKG has limited diagnostic value in suspected PE cases due to the non-specific nature of traditional PE-associated EKG changes. However, it remains crucial in evaluating cardiac conditions such as myocardial ischemia in acute PE scenarios [23,24].

Table 1 presents aggregated data from various studies showing ORs for short-term mortality and adverse events associated with elevated cardiac biomarkers in patients with PE.

**Table 1:** ORs for short-term mortality and adverse events associated with elevated cardiac biomarkers in pulmonary embolism patients. OR = odds ratio; CI = confidence interval; BNP = B-type natriuretic peptide; NT-proBNP = N-terminal pro-brain natriuretic peptide; cTnI = cardiac troponin I; cTnT = cardiac troponin T; hsTnT = high-sensitivity troponin T

Author	Biomarker	OR for short-term mortality	OR for adverse clinical events
Coutance et al. [13]	BNP, NT-proBNP	OR = 6.57, 95% CI = 3.11–13.91	OR = 7.47, 95% CI = 4.2–13.15
Klok et al. [14]	BNP, NT-proBNP	OR = 7.6, 95% CI = 3.4–17	OR = 6.8; 95% CI = 4.4–10
Cavallazzi et al. [10]	BNP	OR = 6, 95% CI = 1.31–27.43	N/A
	NT-proBNP	OR = 16.12, 95% CI = 3.1–83.68	N/A
Becattini et al. [16]	cTnI or cTnT	OR = 5.24, 95% CI = 3.28–8.38	OR = 7.03, 95% CI = 2.42–20.43
Lankeit et al. [17]	hsTnT	N/A	OR = 4.97, 95% CI = 1.71–14.43

## Conclusions

This case report exemplifies the intricate and often challenging nature of diagnosing and managing PE, particularly when it does not present with classic symptoms. The authors underscore the importance of a thorough and holistic assessment in emergencies. In the context of PE, biomarkers such as BNP, NT-proBNP, and troponin, along with a review of the EKG, are pivotal for risk stratification and are expected to become fundamental in future risk assessment tools. Current data supports specific thresholds for cardiac markers in risk assessment in patients with PE. Elevated levels of cardiac biomarkers in PE patients significantly increase the risks of mortality and complications, necessitating further evaluation with echocardiography. Despite its limited role in diagnosing PE due to non-specific changes, EKG is crucial for evaluating conditions such as myocardial ischemia and is vital for risk stratification, especially during the recovery phase, as AF is linked to higher mortality and worse outcomes.
